# C-terminal region of apoptin affects chicken anemia virus replication and virulence

**DOI:** 10.1186/s12985-017-0713-9

**Published:** 2017-02-21

**Authors:** Yongqiang Wang, Xiuqing Song, Honglei Gao, Xiaoyan Wang, Yonghao Hu, Yulong Gao, Xiaole Qi, Liting Qin, Huan Lin, Li Gao, Shuai Yao, Chunyan Han, Xiaomei Wang, Hualan Chen

**Affiliations:** 1 College of Veterinary Medicine, Gansu Agricultural University, Lanzhou, People’s Republic of China; 2 State Key Laboratory of Veterinary Biotechnology, Harbin Veterinary Research Institute, Chinese Academy of Agricultural Sciences, Harbin, People’s Republic of China

**Keywords:** Chicken anemia virus, Apoptin, Apoptosis, Replication, Virulence

## Abstract

**Background:**

Chicken anemia virus (CAV) causes anemia and immune suppression, which are important diseases in the poultry industry. CAV VP3, also referred as ‘apoptin’, has been shown to selectively kill tumor cells, raising great hopes for its utilization as an anticancer therapy. The ability of apoptin to induce apoptosis is closely related to its nuclear localization. The C-terminal region of apoptin contains a bipartite nuclear localization signals (NLS), and a nuclear export signal (NES) is located between the arms of the NLS. Most previous studies have expressed apoptin of different lengths in vitro to understand the relationship between its localization and its induction of apoptosis.

**Methods:**

In this study, we investigated the replication of CAV and its induction of apoptosis in vitro and in vivo with VP3-truncated infectious virus. Quantitative PCR was used to detect viral replication in MDCC-MSB1 cells, and the viral localization was observed by confocal microscopy. Flow cytometry was uesed to analyze virus-induced apoptosis in MDCC-MSB1 cells. Additionally, chickens infected with the rescued viruses compared with the parental virus rM9905 to evaluate the viral replication in vivo and virulence.

**Results:**

Based on the infectious clone, we rescued two viruses in which were deleted NES–NLS2 (rCAV-VP3N88) or NLS1–NES–NLS2 (rCAV-VP3N80) in the C-terminal region of apoptin. The viral load of rCAV-VP3N88 decreased significantly between 60 and 108 hpi, and was always 10–100-fold lower than that of the parental virus rM9905. The levels of rCAV-VP3N80 were also 10–100-fold lower than that of rM9905 and declined significantly at three time points. There was almost no difference in the viral loads of rCAV-VP3N88 and rCAV-VP3N80. Additionally, rM9905 induced 85.39 ± 2.18% apoptosis at 96 hpi, whereas rCAV-VP3N88 and rCAV-VP3N80 induced 63.08 ± 4.78% and 62.56 ± 7.35% apoptosis, respectively, which were significantly (about 20%) lower than that induced by the parental virus. The rescued viruses altered the nuclear localization in MDCC-MSB1 cells. Moreover, deletion of C-terminal region of apoptin impaired viral replication in vivo and reduced the virulence of CAV in chickens.

**Conclusions:**

In summary, we have demonstrated that the C-terminal deletion of apoptin in infectious CAV affected the replication of the virus. The deletion of the C-terminal region of apoptin not only significantly reduced viral replication in vitro but also reduced its induction of apoptosis, which correlated with the loss of its nuclear localization. The deletion of the C-terminal region of apoptin also impaired the replication of CAV and attenuated its virulence in chickens.

## Background

Chicken anemia virus (CAV) causes anemia and immunosuppression, which are important diseases in the poultry industry worldwide. A previous study reported that the *vp3* gene was expressed 12 h after the CAV infection of MDCC-MSB1 cells, whereas the expression of the *vp1* gene was detected at 30 h postinfection. The expression of the *vp3* gene at an early stage of infection suggests that VP3 is involved in viral replication [1]. However, later studies using inhibitors found that VP3 is not involved in de novo gene transcription or translation, and that VP3 itself has no significant transcriptional repression activity, suggesting that VP3 functions in other pathways [2]. The relationship between CAV replication and VP3 in MDCC-MSB1 cells was investigated with VP3 mutants. That study suggested that apoptin is essential not only for DNA replication but also in the virus-like particles of CAV [3]. Therefore, the relationship between VP3 and viral replication requires further investigation.

VP3, also referred as apoptin, is the main virulence factor of CAV and can induce cellular apoptosis [4]. Studies have shown that the ability of apoptin to induce apoptosis is closely related to its nuclear localization. Apoptin specifically induces the apoptosis of tumor and transformed cells, but does not induce the apoptosis of normal diploid cells [5–7]. The C-terminal region of apoptin contains a bipartite nuclear localization signals (NLS) [8], which is necessary for its nuclear accumulation, as shown with analyses of deletion and point mutants [2, 5, 9–11]. However, NLS itself is insufficient for the function of apoptin because the fusion of NLS-mutated apoptin to an external nuclear localization sequence rescued its nuclear localization, but the fused mutant could not induce apoptosis [5]. A exportin-recognized nuclear export signal (NES) that is inactive in tumor cells contributes to the specific localization of apoptin in tumor cells [9]. The NES is located between the arms of the bipartite NLS, so amino acids (aa) 74–121, encompassing both the apoptin NLS and NES, is a tumor cell-specific nuclear targeting signal. Intriguingly, truncated apoptin (aa 74–121) binds to importin β1 with 3-fold greater affinity than does full-length apoptin (aa 1–121), suggesting that the NLS activity of apoptin may be regulated by intramolecular masking by the apoptin N-terminal region [12]. These studies indicate that both NLS and NES play important roles in the nuclear localization of apoptin and therefore its induction of apoptosis. However, most studies have been based on in vitro protein expression analyses, so the functions of modified apoptin within the infectious virus require further investigation.

In this study, we modified the apoptin protein directly in infectious CAV and studied the effects on viral replication and its induction of apoptosis in MDCC-MSB1 cells. We also determined the effects on viral replication and CAV virulence in chickens.

## Methods

### Virus strains, cell lines, and plasmids

CAV strain M9905 was isolated and stored in our laboratory. The pBluescript II SK(+) vector and MDCC-MSB1 cell line are maintained in our laboratory.

### Construction and characterization of pBluemCAV

Primers IU and IL were designed based on the sequence of CAV. The nucleotide at position 1902 was changed from G to A with specific primers to introduce a *Kpn*I restriction site without changing the amino acid sequence. The PCR product was amplified using primers IU and IL and the genomic DNA of CAV as template. After purification, the PCR product was digested with *Kpn*I. The pBluescript II SK(+) vector was digested with *Kpn*I and dephosphorylated with phosphatase. After purification, the vector was ligated to the *Kpn*I-digested genomic DNA, and the ligation product was used to transform competent *Escherichia coli* DH5α cells. A single colony was picked and the plasmid was isolated. The plasmid was characterized with PCR, *Kpn*I digestion, and sequencing.

### Construction of mutant plasmids

**Fig. 1:**
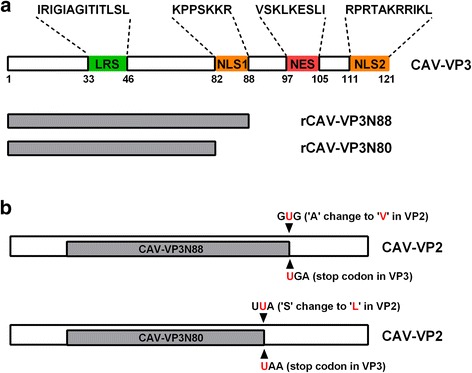
Schematic representation of CAV VP3 with different deletions and changes to VP2 (not drawn to scale). **a** A leucine-rich sequence (LRS) is located at residues 33–46. Nuclear localization signal 1 (NLS1) is located at residues 82–88. Nuclear export signal (NES) is located at residues 97–105. NLS2 is located at residues 111–121. Rescued virus rCAV-VP3N88 is deleted at NES–NLS2 and rescued virus rCAV-VP3N80 is deleted at NLS1–NES–NLS2. **b** The stop codon of ‘UGA’ is introduced in VP3 to construct rCAV-VP3N88. However, this stop codon will not introduced into VP2 because they are in different frames. And only making an amino acid substitution of ‘A’ with a ‘V’ in VP2. Similarly, the stop codon of ‘UAA’ is introduced in VP3 to construct rCAV-VP3N80. And resulting an amino acid substitution of ‘S’ with a ‘L’ in VP2. These VP3 truncated mutant viurses express the full length VP2 as the parental virus

To investigate the effect of VP3 on viral replication, we constructed VP3 deletion mutants of the virus and determined the regions within VP3 that are essential for viral replication. Primers pairs 88U/88 L and 80U/80 L were designed (Table 1). A stop codon was introduced at different positions in the *vp3* gene to terminate the translation of VP3 prematurely [13]. Briefly, a stop codon was introduced with PCR using primers 88U/88 L or 80U/80 L, with pBluemCAV as the template, and with PrimeSTAR™ HS DNA polymerase under the following conditions: pre-denaturation at 95 °C for 5 min and 20 cycles of 95 °C for 30 s, 55 °C for 1 min, 72 °C for 6 min, followed by 72 °C for 7 min. The PCR products were precipitated with absolute ethanol, then dissolved in ddH_2_O, and digested with DpnI for 1 h at 37 °C in order to remove DNA of the parent plasmid pBluemCAV. The digested PCR products were used to transform Competent E. coli cells and 12 single colonies were picked to isolate the plasmids. Specific primers Utu/Ltu were used to amplify the vp3 gene for sequencing. Plasmids with the correct point mutations were sent for whole-genome sequencing. Thus, the amino acids from position 88 or 80 to position 121 of VP3 were deleted, and an amino acid substitution was introduced in VP2 but without influence on expression of full length of VP2 (Fig. 1). Based on previous studies, we ligated two copies of the CAV genomic DNA into the pBluescript II SK(+) vector to construct an infectious plasmid [14–17]. Positive plasmids with the correct point mutations were partially digested with *Kpn*I, dephosphorylated, and ligated to the genomic DNA. Competent *E. coli* DH5α cells were transformed with the ligation products. The plasmids were sequenced to confirm that the genomic DNA was successfully ligated to the vector in the correct orientation. The plasmids with point mutations in the *vp3* gene were designated pBluem2CAV-VP3NX.

**Table 1:** Primer sequences used in this study^Note^

Name	Sequences	The position with point mutation
88U	5’-CTCCCTCGAAGAAG**T**GATCCTGCGACCCCTC-3’	747
88 L	5’-GAGGGGTCGCAGGATC**A**CTTCTTCGAGGGAG-3’	747
80U	5’-CTTGAGGACCGAT**T**AACCCAAGCCTCCCTCG-3’	723
80 L	5’- CGAGGGAGGCTTGGGTT**A**ATCGGTCCTCAAG-3’	723
Utu	5’-CATACCGGTCGGCAGTAG-3’	-
Ltu	5’-CGATACCGCTGTCTCCTC-3’	-
IU	5’-GCTGGT **A** CCGCTCGGCACGGAGAC-3’	1902
IL	5’-AGGGG **T** ACCAGCGTGTGCCATCTC-3’	1902

^Note^Mutated nucleotides were in bold in the primer sequences, and the restriction enzyme sites were underlined

### Virus rescue and characterization

The plasmids were isolated from 100 mL cultures of positive *E. coli* clones and dissolved in 100 μL of ddH_2_O. After quantification, the plasmids were stored at −20 °C for future use. Cells were electrotransfected with the plasmids at 375 V and 800 μF. The transfected cells were passage once every 2 days. The cells were centrifuged, the old medium discarded, and the cells resuspended in three volumes of medium for further culture. An immunofluorescence assay was performed as described previously [18]. Briefly, a primary monoclonal antibody against VP3, diluted 1:200, was used. The secondary antibody was a fluorescein isothiocyanate (FITC)-conjugated rabbit anti-mouse IgG antibody, diluted 1:200. The virus was detected in the cells with PCR at each passage. The cells were collected and the viral genomic DNA extracted. The rescued virus was detected with PCR using primers Utu/Ltu and confirmed with sequencing. The viral proteins VP2 and VP3 were detected with monoclonal antibody against VP2 or VP3, respectively, in western blotting assay. Cells were washed with phosphate buffered saline (PBS) and were lysed with lysis buffer containing 20 mM Tris-HCl pH 7.5, 150 mM NaCl, 1% Triton X-100, sodium pyrophosphate, β-glycerophosphate, and 1× complete cocktail protease inhibitor. Whole lysates were boiled for 10 min in the presence of 5× SDS-PAGE loading buffer. After centrifugation at 12,000 × g for 2 min, equivalent sample amounts were separated by 12% SDS-PAGE and transferred to pure nitrocellulose blotting membranes. After blocking with 5% skim milk, the membranes were incubated with primary antibody at 37 °C for 1.5 h, followed by HRP-conjugated Anti-Mouse secondary antibody for 1 h at 37 °C. Proteins were visualized by enhanced chemiluminiscence (ECL) using the CLiNX Science Instruments.

### Quantitative PCR to detect viral replication in MDCC-MSB1 cells

The viruses with point mutations in the *vp3* gene were rescued and designated rCAV-VP3N88 and rCAV-VP3N80. Their replication was quantified and compared with that of the parental strain rM9905 using quantitative PCR. Viral replication was determined at 48, 60, 72, 84, 96, and 108 h after the inoculation of MDCC-MSB1 cells. Briefly, the viral genomic DNA was isolated from 1 mL of virus solution and dissolved in 20 μL of sterile water. Quantitative PCR was performed to determine the copies of virus present. The upstream and downstream primers for quantitative PCR were 5′-AATTTCGACATCGGAGGAG-3′ and 5′-GGAAGCGGATAGTCATAGTAGAT-3′, respectively. The probe sequence was 5′-FAM-AGCGGTATCGTAGACGAGCTTTTAGGAAGGC-TAMRA-3′. The reaction volume was 20 μL, containing 2.0 μL of 10 × buffer, 2.0 μL of dNTPs (2.5 μmol/L), 0.5 μL each of the upstream and downstream primers (10 μmol/L), 0.3 μL of probe (10 μmol/L), 0.12 μL of Hot Start *Taq* DNA Polymerase (5 U/μL), 2 μL of template DNA, and the appropriate amount of ddH_2_O. The cycling parameters for quantitative PCR were predenaturation at 95 °C for 3 min followed by 40 cycles of 94 °C for 15 s, 56 °C for 20 s, and 72 °C for 20 s.

### Confocal microscopic localization of virus in MDCC-MSB1 cells

The localization of the NLS-deleted viruses rCAV-VP3N88 and rCAV-VP3N80 was determined and compared with that of the parental virus rM9905 in MDCC-MSB1 cells using confocal microscopy. The viruses were labeled with a mouse primary antibody directed against VP3 and a secondary FITC-conjugated rabbit anti-mouse IgG antibody. The cell nuclei were stained with propidium iodide (PI). Cells infected with rM9905 were used as the positive control and uninfected cells were used as the negative control.

### Flow-cytometric analysis of virus-induced apoptosis in MDCC-MSB1 cells

Apoptosis was detected with flow cytometry. Cells at the concentration of 10^5^/mL were infected with rCAV-VP3N88, rCAV-VP3N80, or rM9905. Uninfected cells were used as the negative control. At 48, 72, and 96 h after inoculation, apoptosis was detected with an Annexin V–FITC Apoptosis Detection Kit (BD, San Jose, CA).

### Animal experiments

The animal experiments were performed in strict compliance with the Guideline for the Care and Use of Laboratory Animals of the Ministry of Science and Technology of the People’s Republic of China. The animal experiment protocols were also approved by the Committee of the Ethics of Animal Experiments at the HVRI, CAAS. The legs and neck muscles of chickens were inoculated at multiple sites with the viral strains (rCAV-VP3N88, rCAV-VP3N80, or rM9905) at a concentration of 10^5^ copy/mL. One-day-old specific-pathogen-free (SPF) chickens (18 chickens allocated randomly to each group) were inoculated with 200 μL of viral solution or RPMI 1640 medium, and their clinical symptoms were recorded. The chickens were euthanized at 5, 9, 11, 14, 21, and 28 days postinfection (dpi). Their tissues were collected for histological analysis, DNA sequencing, and quantitative PCR analysis. The thymuses were collected and the viral genomic DNA extracted. A virus replication curve was constructed based on the load of viral DNA determined with quantitative PCR, as described above. Primers Utu/Ltu were used for the PCR reaction. The PCR products were purified and sent for sequencing to confirm the presence of the appropriate point mutations.

### Statistical analysis

All data are presented as the means ± SD of three independent experiments. The significance of the variations between groups was determined with a *t* test (#*p* > 0.05; *p < 0.05; **p < 0.01).

## Results

### Rescue and characterization of CAV

**Fig. 2:**
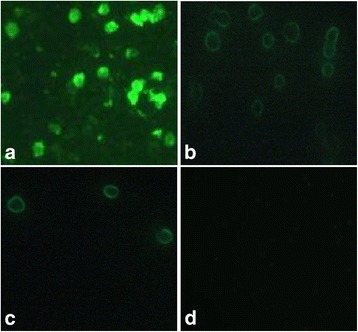
Immunofluorescence assay of the rescued viruses. MDCC-MSB1 cells were infected with the parental virus rM9905 (**a**) or rescued viruses rCAV-VP3N88 (**b**) and rCAV-VP3N80 (**c**), or were mock-infected (**d**). Cells were treated with primary antibody directed against VP3 and FITC-conjugated secondary antibody, and detected with microscopy (×200)

To directly investigate the functions of the NLS1, NES, and NLS2 domains in viral replication, virulence, and the nuclear localization of CAV, viruses with deletions in the C-terminal region of apoptin were rescued and characterized both in vitro and in vivo. An immunofluorescence assay showed that the rescued virus could be detected with a monoclonal antibody directed against CAV (Fig. 2). Sequence analyses showed that point mutations were correctly introduced into the *vp3* gene, confirming that the viruses with deletions in the VP3 protein were successfully rescued. The viruses in which were deleted NES–NLS2 of apoptin (rCAV-VP3N88) or NLS1–NES–NLS2 of apoptin (rCAV-VP3N80) mainly localized to the cytosol (Fig. 2). This clearly differed from the distribution pattern of the parental virus rM9905, which localized throughout the whole cell.

**Fig. 3:**
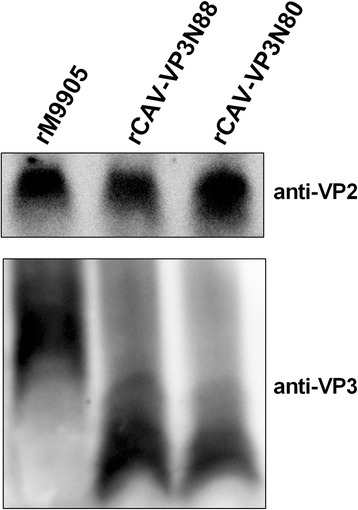
Viral proteins VP2 and VP3 expressed in MDCC-MSB1 cells infected with rCAV-VP3N88, rCAV-VP3N80, or parental virus rM9905, respectively. Whole cell lysates were separated by SDS-PAGE and transferred to pure nitrocellulose blotting membranes. Monoclonal antibodies against VP2 or VP3 were used as primary antibody, followed by HRP-conjugated Anti-Mouse secondary antibody. Proteins were visualized by ECL assay

Viral proteins both VP2 and VP3 were evaluated by western blotting in VP3 truncated viruses compared with parental virus. As shown in Fig. 3, the parental virus rM9905 possess the entire VP3 with 121 amino acids, which showed about 14 kDa protein in WB assay. The virus rCAV-VP3N88 with a truncated VP3 of 88 amino acids showed about 10 kDa protein. And the virus rCAV-VP3N80 with a truncated VP3 of 80 amino acids showed about 9 kDa protein in WB. These results showed that the monoclonal antibody against VP3 could identify both the entire VP3 and the truncated VP3 with only 88 or 80 amino acids in N-terminus of protein. The parental virus expressed relative high amount of VP3, and mutant viruses expressed truncated VP3. Besides, the almost same molecular weight (~24 kDa) of VP2 was showed in rCAV-VP3N88, rCAV-VP3N80, and rM9905, which indicated that VP3 truncated virus did not influence the expression of viral VP2.

### Deletion of C-terminal region of apoptin reduced viral replication in MDCC-MSB1 cells

**Fig. 4:**
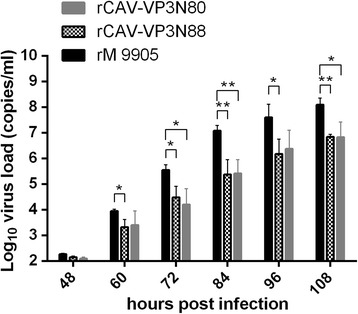
Replication of rescued viruses rCAV-VP3N88 and rCAV-VP3N80 compared with parental strain rM9905 in MDCC-MSB1 cells. MDCC-MSB1 cells were inoculated with the same amounts of virus, quantified with quantitative PCR. Viral replication was determined with quantitative PCR at 48, 60, 72, 84, 96, and 108 h postinfection. The error bars represent the standard deviations at each time point for triplicate assays. Significance of the variations between groups was determined with a *t* test (#*p* > 0.05; *p < 0.05; **p < 0.01)

To study the effects of the deletion of the C-terminal region of apoptin on viral replication in vitro, the viral loads of rCAV-VP3N88, rCAV-VP3N80, and rM9905 were determined with quantitative PCR. As shown in Fig. 4, the viral load increased steeply from 48 to 84 h postinfection (hpi), reaching a plateau phase at 96 hpi. The time courses of replication for rCAV-VP3N88 and rCAV-VP3N80 were similar to that of the parental virus rM9905, but their viral loads were lower at each time point. The viral load of rCAV-VP3N88 decreased significantly between 60 and 108 hpi, and was always 10–100-fold lower than that of the parental virus rM9905. The levels of rCAV-VP3N80 were also 10–100-fold lower than that of rM9905 and declined significantly at three time points. There was almost no difference in the viral loads of rCAV-VP3N88 and rCAV-VP3N80. These results indicate that the replication capacity of CAV deleted in the C-terminal region of apoptin was significantly reduced in vitro.

### Deletion of C-terminal region of apoptin alters viral nuclear localization

**Fig. 5:**
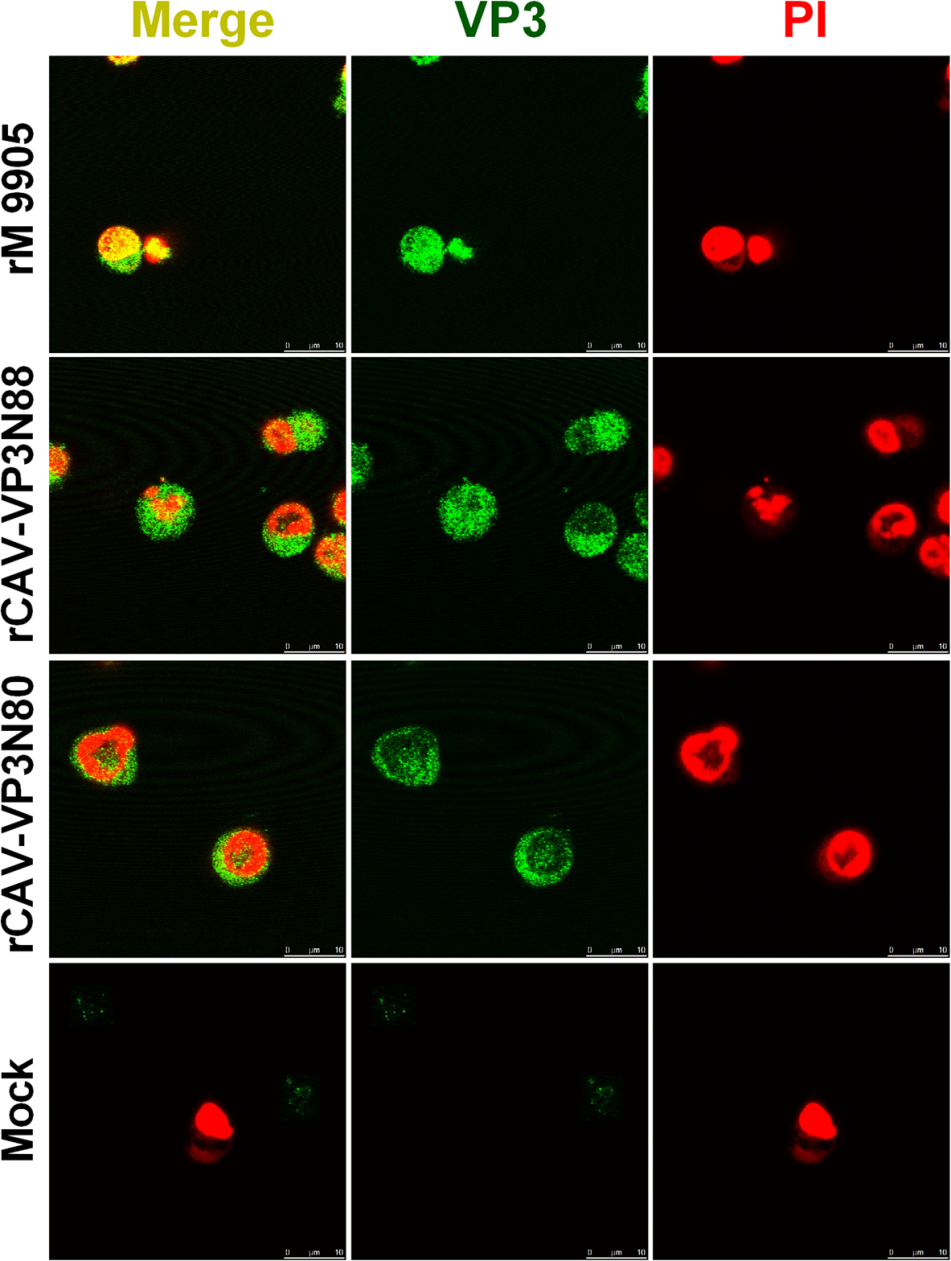
Localization of rescued viruses rCAV-VP3N88 and rCAV-VP3N80 compared with parental strain rM9905 in MDCC-MSB1 cells. MDCC-MSB1 cells were infected with rescued virus rCAV-VP3N88 or rCAV-VP3N80 or parental virus rM9905. Mock-infected cells were used as the negative control. Viruses were stained with FITC-conjugated antibody (*green*) and nuclei with propidium iodide (PI; *red*). The distribution of apoptin was observed with confocal microscopy. Scale bar is shown at the *bottom right* (10 μm)

To observe the distribution of the viruses with deletions in the C-terminal regions of apoptin, confocal microscopy was used to determine viral localization in MDCC-MSB1 cells after the viruses were labeled with green fluorescence (FITC) and the cell nuclei with red fluorescence (PI). As shown in Fig. 5, viruses with the NES–NLS2 deletion (rCAV-VP3N88) or the NLS1–NES–NLS2 deletion (rCAV-VP3N80) were distributed in the cytosol, because the green fluorescent signal was mainly detected in the cytosol, whereas the nucleic were predominantly stained red. In contrast, the parental virus rM9905 localized to the nuclei, appearing as a yellow signal, produced when the green viral signal and red nuclear signal merged. The uninfected cells stained red in their nuclei. These results indicate that the deletion of the C-terminal region of apoptin alters the nuclear localization of the virus in MDCC-MSB1 cells.

### Deletion of C-terminal region of apoptin reduces the capacity of CAV to induce apoptosis

**Fig. 6:**
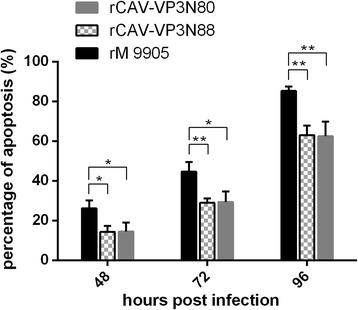
Apoptosis induced by rescued viruses rCAV-VP3N88 and rCAV-VP3N80 compared with that induced by parental strain rM9905 in MDCC-MSB1 cells. MDCC-MSB1 cells were infected with rescued virus rCAV-VP3N88 or rCAV-VP3N80 or parental virus rM9905. At 48, 72, and 96 h postinfection, apoptosis was detected with an annexin V–FITC apoptosis detection kit and flow cytometry. Error bars represent the standard deviations at each time point for triplicate assays. Significance of the variations between groups was determined with a *t* test (#*p* > 0.05; *p < 0.05; **p < 0.01)

To investigate the effects of C-terminal deletions of apoptin on virus-induced apoptosis, apoptosis was detected with flow cytometry using an annexin V–FITC detection kit. The apoptosis induced by the viruses increased between 48 and 96 hpi, and the parental virus rM9905 induced a higher proportion of apoptotic cells than either rCAV-VP3N88 or rCAV-VP3N80 at every time point (Fig. 6). For example, rM9905 induced 85.39 ± 2.18% apoptosis at 96 hpi, whereas rCAV-VP3N88 and rCAV-VP3N80 induced 63.08 ± 4.78% and 62.56 ± 7.35% apoptosis, respectively, which were significantly (about 20%) lower than that induced by the parental virus (Fig. 6). These results indicate that the deletion of the C-terminal region of apoptin reduces the capacity of CAV to induce apoptosis.

### Deletion of C-terminal region of apoptin impairs viral replication in vivo

**Fig. 7:**
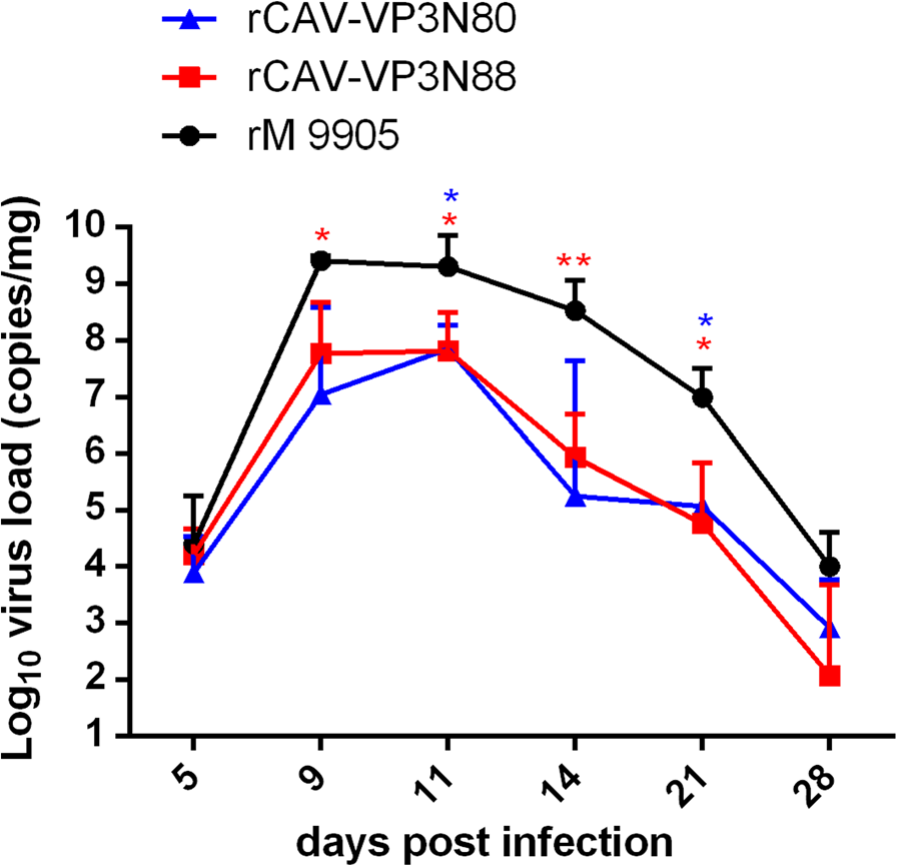
Replication of rescued viruses rCAV-VP3N88 and rCAV-VP3N80 compared with parental strain rM9905 in thymuses of SPF chickens. SPF chickens were inoculated with rescued virus rCAV-VP3N88 or rCAV-VP3N80 or parental virus rM9905. The viral replication curve was constructed from the viral DNA loads in the thymus, measured with quantitative PCR. Error bars represent the standard deviations at each time point for triplicate assays. Significance of the variations between groups was determined with a *t* test (#*p* > 0.05; *p < 0.05; **p < 0.01)

Preliminary studies showed that the thymus has the highest load of virus after infection. Therefore, to evaluate the effect of the deletion of the C-terminal region of apoptin on viral replication in vivo, we used the viral load in the chicken thymus as a measure of viral replication in vivo. The viral load was quantified with quantitative PCR. The replication kinetics of rCAV-VP3N88 and rCAV-VP3N80 were similar to those of rM9905, but with lower viral loads. The viral load increased dramatically at 5–9 dpi. The highest viral load in the chicken thymus occurred at 9–11 dpi and decreased gradually thereafter (Fig. 7). The viral loads in the thymuses of chickens infected with rCAV-VP3N88 or rCAV-VP3N80 were 10–100-fold lower than those in chickens infected with the parental strain rM9905 at 9–21 dpi. The viral load of rCAV-VP3N88 was significantly lower than that of rM9905 at 9, 11, 14, and 21 dpi, and the viral load of rCAV-VP3N80 was significantly lower than that of the parental strain at 11 and 21 dpi (Fig. 7). These results indicate that the deletion of C-terminal region of apoptin impairs viral replication in vivo.

### Deletion of the C-terminal region of apoptin reduces CAV virulence in chickens

**Fig. 8:**
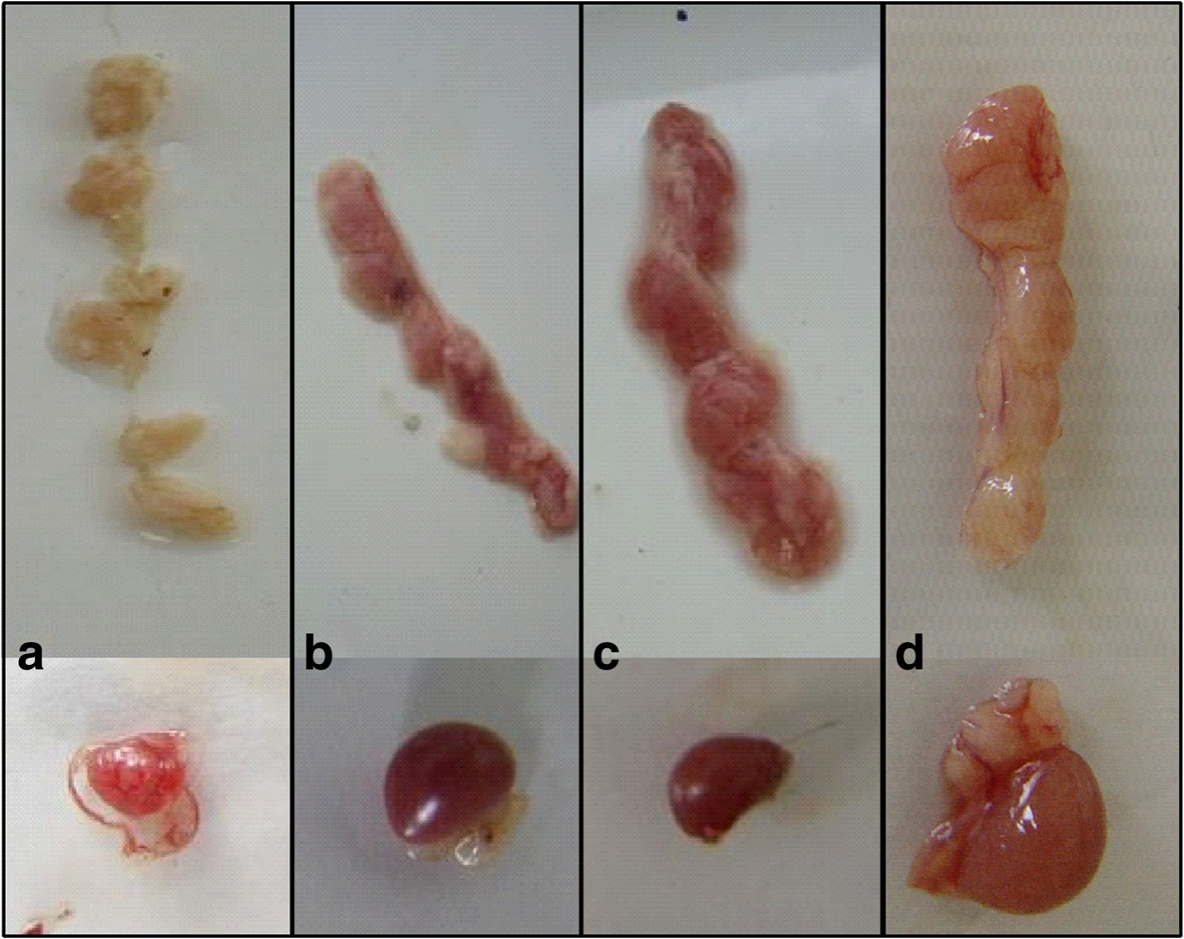
Gross changes in the thymuses and spleens of SPF chickens infected with rM9905 (**a**), rCAV-VP3N88 (**b**), rCAV-VP3N80 (**c**), or mock-infected (**d**)

**Fig. 9:**
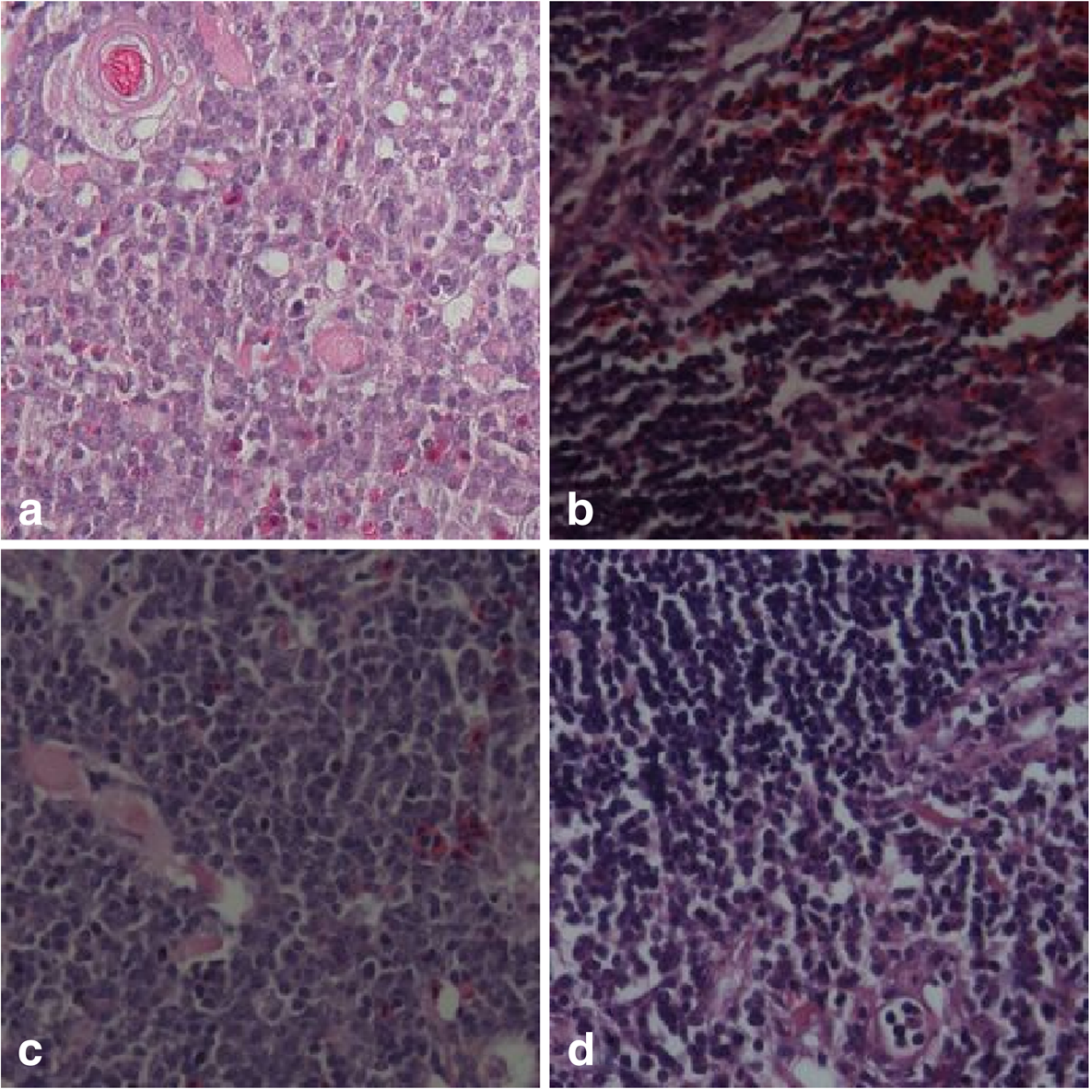
Histological changes in the thymuses of SPF chickens infected with rM9905 (**a**), rCAV-VP3N88 (**b**), rCAV-VP3N80 (**c**), or mock-infected (**d**)

An autopsy analysis showed that chickens infected with the parental virus rM9905 for 5, 9, 11, 14, 21, or 28 days suffered severe anemia (anemia was most obvious at 9–11 dpi), and hemorrhage occurred in the leg and chest muscles (data not shown). The thymus showed hemorrhage or severe atrophy (Fig. 8). The chickens infected with rCAV-VP3N88 or rCAV-VP3N80 had only mild hemorrhage in the leg and chest muscles and the thymus. The thymuses displayed no obvious changes in the uninfected chickens (Fig. 8). Histological examinations were performed on three chickens from each group. In chickens infected with the parental strain, the volume of the thymus lobule was reduced, the number of lymphocytes was reduced, hemorrhage and congestion were apparent, and medulla atrophy was present (Fig. 9). In the chickens inoculated with rCAV-VP3N88 or rCAV-VP3N80, the medulla and cortex displayed hemorrhage and congestion and the vessels showed blood stasis. There were no obvious histological changes in the thymuses of the uninfected chickens. These results indicate that the deletion of the C-terminal region of apoptin reduces the virulence of CAV in chickens.

## Discussion

In this study, we investigated the replication of CAV and its induction of apoptosis in vitro and its replication and virulence in vivo, using infectious virus with truncated VP3. Apoptin has two NLSs. NLS1 is located at amino acids 82–88, and NLS2 is located at amino acids 111–121; NLS1 and NLS2 are interdependent. The NES (aa 97–105) is located between the arms of the bipartite NLS. We constructed and rescued two mutant viral strains rCAV-VP3N88 (lacking NES–NLS2) and rCAV-VP3N80 (lacking NLS1–NLS2). Compared with the parental virus, the deletion of the C-terminal region of apoptin by removing either NES–NLS2 or NLS1–NES–NLS2 significantly reduced the replication of CAV both in vitro and in vivo. The virus in which apoptin was completely deleted could not be rescued (data not shown). Therefore, apoptin is involved in CAV replication. However, the replication kinetics of the strains lacking NES–NLS2 or NLS1–NES–NLS2 were almost identical, indicating that NLS1 alone cannot alter the replication of the virus significantly in a backbone already lacking NES–NLS2. A confocal microscopy analysis showed that the viruses lacking NES–NLS2 or NLS1–NES–NLS2 localized mainly to the cytosol, differing from the nuclear localization of the parental virus. These results indicate that the effect of apoptin on viral replication correlates with the nuclear localization of CAV. The NLS1 alone could not ensure the nuclear localization of the virus in a backbone already lacking NES–NLS2. The subcellular localization of apoptin is controlled by several competing processes within the cell, including its nuclear and cytoplasmic retention and facilitated nuclear import and export. NES contributes to the tumor-cell-specific localization of apoptin [9]. Apoptin threonine 108 (T108), adjacent to NES, is specifically phosphorylated by an unidentified kinase in tumor cells but not in normal cells [19]. Phosphorylation at this site appears to be responsible for the tumor-cell-specificity of apoptin nuclear localization, by inhibiting its NES-mediated nuclear export in tumor cells [9] and possibly also by enhancing its nuclear import [19, 20]. Apoptin has also been reported to bind other nuclear components, such as the homeodomain-interacting protein kinase 2 (HIPK2) [9], human death effector domain-associated factor [21], DNA [22], and APC-1, a subunit of the anaphase-promoting complex/cyclosome, which is involved in the assembly and regulation of the cyclosome complex [23]. VP3 has also been reported to interact with the cytoplasmic protein HIPPI, a protein that interacts with Huntingtin interacting protein 1 [24].

To determine the relationship between the nuclear localization of apoptin and apoptosis, Noteborn and his partners coexpressed both apoptin and the large T antigen of SV40 in normal cells and found that apoptin localized into the nuclei and induced the apoptosis of cells transformed with the large T antigen [25]. Apoptin also localized to the nuclei and induced the apoptosis of UV-exposed skin diploid fibroblast cells derived from humans with an inherited cancer susceptibility syndrome. In contrast, apoptin mainly localized to the cytosol and did not induce apoptosis in UV-exposed skin diploid fibroblast cells derived from normal humans. These studies indicate that the nuclear localization of apoptin correlates with the induction of apoptosis [7]. The protein sequences at aa 100–121, aa 90–121, aa 80–121, aa 70–121 and aa 1–69 in the VP3 protein were expressed in Saos-2 cells, and showed that the N-terminus or C-terminus of VP3 alone is sufficient to induce apoptosis, but the apoptosis-inducing activities of these truncated proteins were lower than that of full-length VP3 [2]. A previous study investigated the effects of p53 on the induction of apoptosis by apoptin and found that p53 did not affect its apoptotic activity. Full-length apoptin induced 80%–90% apoptosis, whereas apoptin with deleted NLS2 induced only 40% apoptosis 6 days after transfection, when the apoptosis rate was calculated by counting 100 PI-stained cells in one microscope field [26]. In this study, we used flow cytometry to determine the apoptosis rate in 10,000 cells. The apoptosis-inducing ability of the virus lacking either NES–NLS2 or NLS1–NES–NLS2 was 20% lower than that of the parental virus. Furthermore, the apoptosis-inducing ability of the virus with the NLS1–NES–NLS2 deletion was similar to that with the NES–NLS2 deletion. Therefore, these two viruses may have almost lost their ability to localize to the nucleus, and be similarly distributed in cells.

## Conclusions

In summary, we have demonstrated that the C-terminal deletion of apoptin in infectious CAV affected the replication of the virus. The deletion of the C-terminal region of apoptin not only significantly reduced viral replication in vitro but also reduced its induction of apoptosis, which correlated with the loss of its nuclear localization. The deletion of the C-terminal region of apoptin also impaired the replication of CAV and attenuated its virulence in chickens.

## Abbreviations

APC-1: Subunit of the anaphase-promoting complex/cyclosome
CAV: Chicken anemia virus
FAM: Carboxyfluorescein
FITC: Fluorescein isothiocyanate
HIPK2: Homeodomain-interacting protein kinase 2
HIPPI: Protein interactor of the Huntingtin interacting protein 1
LRS: Leucine-rich sequence
NES: Nuclear export signal
NLS: Nuclear localization signal
PCR: Polymerase chain reaction
PI: Propidium iodide
RPMI: Roswell park memorial institute
SPF: Specific-pathogen-free
TAMRA: Carboxytetramethylrhodamine
VP3: Viral protein 3
